# Evaluation of the Content of Minerals, B-Group Vitamins, Tocols, and Carotenoids in Raw and In-House Cooked Wild Edible Plants

**DOI:** 10.3390/foods13030472

**Published:** 2024-02-02

**Authors:** Alessandra Fratianni, Donatella Albanese, Giuseppe Ianiri, Caroline Vitone, Francesca Malvano, Pasquale Avino, Gianfranco Panfili

**Affiliations:** 1 Department of Agricultural, Environmental and Food Sciences, University of Molise, Via De Sanctis, 86100 Campobasso, Italy; g.ianiri@studenti.unimol.it (G.I.); c.vitone@studenti.unimol.it (C.V.); avino@unimol.it (P.A.); panfili@unimol.it (G.P.); 2 Department of Industrial Engineering, University of Salerno, Via Ponte Don Melillo, 84084 Fisciano, Italy; dalbanese@unisa.it (D.A.); fmalvano@unisa.it (F.M.)

**Keywords:** wild edible plants, vitamin activity, tocols, carotenoids, B-group vitamins, minerals, boiling, steaming

## Abstract

Notwithstanding the increased interest in wild edible plants, little is known on how some domestic thermal processes can affect their content. The aim of this study was to investigate the amounts of minerals, B1 and B2 vitamins, tocols, and carotenoids in raw, boiled, and steamed wild edible plants, namely, *Sonchus asper* (L.) Hill s.l., *Sonchus oleraceus* L., *Cichorium intybus* L., and *Beta vulgaris* L. var *cicla*. All vegetables were confirmed as high sources of lutein (from 6 to 9 mg/100 g) and β-carotene (from 2 to 5 mg/100 g). Quite high amounts of violaxanthin and neoxanthin were found. Alfa-tocopherol and γ-tocopherol were the main tocols, with same contents in raw and processed vegetables (about 2.5 mg/100 g). The most abundant macro element and trace element were, respectively, potassium and iron. B1 and B2 vitamins were found in low amounts in almost all plants, with the exception of thiamine in *Beta vulgaris* (about 1.6 mg/100 g). Boiling led to a significant loss of minerals (up to 60%) and B-group vitamins (up to 100%), while, among carotenoids, it only affected violaxanthin levels (up to 90%). Steamed vegetables showed only a slight reduction, about 20%, in β-carotene and lutein, with a marked decrease in violaxanthin and neoxanthin. One hundred grams of all fresh and cooked plants can be claimed as a source of vitamin A and E.

## 1. Introduction

The human body cannot produce minerals and vitamins; therefore, they need to be supplied by food. Wild edible plants (WEPs) have been recognised to have health effects against several chronic disorders [1]. WEPs have high amounts of fibre, proteins, and several minerals and provide high contents of bioactive compounds, such as polyphenols, vitamins, tocols, and carotenoids, which play an important therapeutic and nutritional role [1,2,3,4].

In this regard, some minerals are the main components of bones, while others participate to several life processes. Among B-group vitamins, thiamine (vitamin B1) acts as a co-factor to assist in the activity of different enzymes in wholegrains, cereals, nuts, seeds, liver, and pulses. Riboflavin (vitamin B2) is involved in flavoprotein enzymes and, apart by leafy green vegetables, good amounts are provided by milk products and whole cereals [1,5].

Carotenoids are pigments divided into carotenes and xanthophylls. Carotenes are precursors of retinol, better known as vitamin A. Among xanthophylls, lutein and zeaxanthin are antioxidants. The available evidence suggests that lutein is associated with the reduction of the development of chronic diseases and, even if with controversial results, and has a beneficial impact on the visual function [6,7]. In higher plants, essential photoprotective functions have been assigned to lutein in the heat dissipation of excess light energy [8]. Different carotenoids, such as α-carotene, β-carotene, β-cryptoxanthin, lutein, and epoxycarotenoids, in their free or esterified forms, are provided in the diet through yellow/orange fruits and vegetables, such as green leafy ones [1,5,9,10,11]. 

Tocols are structurally similar compounds. The most common ones are tocopherols, namely, α-tocopherol (α-T), β-tocopherol (β-T), γ-tocopherol (γ-T), and δ-tocopherol (δ-T), and tocotrienols, namely, α-tocotrienol (α-T3), β-tocotrienol (β-T3), γ-tocotrienol (γ-T3), and δ-tocotrienol (δ-T3). According to recent evidence, only α-tocopherol seems to exhibit vitamin E activity and it is still unclear whether other tocol molecules can prevent vitamin E avitaminosis in humans as α-T does [12]. Studies have shown that a dietary intake of tocols helps to prevent the occurrence of neoplasms and heart diseases [12,13]. Foods containing the highest contents of tocols are vegetables, in particular seeds and derived oils [13].

Almost all green leafy vegetables are consumed after different domestic processes, such as boiling, steaming, frying, and microwaving. This leads to the degradation of heat-sensitive compounds and/or the leaching of water-soluble nutrients in the cooking water, with the consequent loss of bioactive constituents, such as carotenoids, polyphenols, and certain vitamins and minerals. The extent of loss depends on the type of vegetable, genotype, structure, compound organization in the food matrix, and cooking methods [14,15,16,17,18]. The degree of these changes may be influenced by temperature and time conditions, oxygen occurrence, water, light, pH, and the presence of other antioxidants [18,19,20]. Some plant enzymes (such as lipoxygenase) also accelerate compound degradation [21]. When subjected to heat treatments, carotenoids and tocols may degrade and thus undergo a disruption of their molecular structure. In particular, the *cis*-isomerization of carotenoids is one of the most frequent reactions [22]. Published papers on fruits and vegetables show the impact of different cooking processes on bioactive compounds, with controversial results. While, in some cases, a decrease in nutrients is found [14,18,23], in others, an increase is reported. This finding is due to the liberation of bound compounds, resulting in a higher bioavailability and extractability [14,15,16,17]. Some research studies attribute these increases to the loss of moisture and the leaching of soluble solids in the cooking water, which concentrate the sample per unit weight, when data are expressed on a dry weight basis [23,24].

In recent years, the attention on WEPs has quickly grown. However, there is still little information on wild plants of popular consumption, especially in terms of nutritional changes caused by domestic cooking. Therefore, the aim of the present work was to investigate the amounts of minerals, vitamin B1 and B2, carotenoids, and tocols of raw and in-house processed wild green leafy vegetables. 

The basal leaves of four WEPs, *Sonchus oleraceus* L., *Sonchus asper* (L.) Hill s.l., *Beta vulgaris* L. var. *cicla*, and *Cichorium intybus* L., were investigated as raw and after boiling and steaming treatments. *Spinacia oleracea* L. was used as a control green leafy vegetable. The contribution to the vitamin activity of the investigated compounds and the percentage of the recommended daily allowance (RDA) were also taken into consideration. 

The findings of this study not only could be of interest for a nutritional evaluation, but also for generating awareness among consumers regarding the extent to which certain common domestic thermal treatments could induce changes in some water-soluble and fat-soluble bioactives.

## 2. Materials and Methods

### 2.1. Plant Material

The basal leaves of four WEPs were investigated: *Sonchus oleraceus* L., *Sonchus asper* (L.) Hill s.l., *Beta vulgaris* L. var. *cicla* (chard), and *Cichorium intybus* L. (chicory). The investigated WEPs were chosen on the basis of their wide diffusion and mode of consumption in the south of Italy, according to [25]. WEPs were collected, during spring, in 2021, in Italy, in particular in the regions of Molise (Ripalimosani-CB) and Campania (Circello-BN). *Spinacia oleracea* L. (spinach) was bought in local markets. For each sample, a minimum of 1 kg was gathered. Damaged parts and foreign bodies were removed, and some leaves were immediately stored at −20 °C in order to be analysed as raw.

### 2.2. Samples Preparation and Cooking Treatments

On fresh leaves of each vegetable two cooking treatments, traditional boiling and steaming, were carried out. The conditions used for cooking were those described in Fratianni et al. [23]. Three repetitions were carried out on each sample, for each type of cooking treatment. One hundred grams of fresh leaves were boiled in 1 L of boiling water (vegetable to water ratio 1:10) for 10 min, according to the common conditions used in the literature [26] and their consumption [23]. Steaming was carried out by placing, on a rack, 100 g of leaves over boiling water, in a closed water bath for 10 min. Aliquots of raw samples, boiled, and steamed samples were freeze-dried (Genesis 25SES freeze-dryer, VirTis Co., Gardiner, NY, USA). The freeze-dried samples were milled using a water-cooled mill (IKA^®^-Werke GmbH & Co. KG, Staufen, Germany), to avoid the overheating of the mass, and stored, under vacuum, at −20 °C until analysis. The moisture of raw, thermally processed, and freeze-dried leaves was determined by measuring weight loss after heating the samples at 130 °C [27].

### 2.3. Chemicals

Solvents, at the highest purity, and other analytical grade reagents were acquired from Sigma (Sigma Aldrich, St. Louis, MO, USA). Alfa-carotene, 9-*cis*-β-carotene, 13-*cis*-β-carotene, violaxanthin, and neoxanthin standards were purchased from CaroteNature (Lupsingen, Switzerland); lutein, zeaxanthin, and β-cryptoxanthin were acquired from Extrasynthese (Z.I. Lyon-Nord, Genay, France). All-trans-β-carotene, thiamine, and riboflavin were purchased from Sigma Chemicals; α, β, γ, and δ-tocopherol standards were acquired from Merck (Darmstadt, Germany); α, β, γ, and δ-tocotrienol standards were obtained as in Panfili et al. [28].

### 2.4. Determination of Minerals

Macro and trace elements were determined in each sample, according to the procedure of Kawashima and Valente Soares [29] with some modifications. Two grams of each sample were weighed into polytetrafluoroethylene digestion vessels, and then 5 mL HNO_3_ (65%) and 1 mL H_2_O were added. The vessels were digested in a microwave digestion system (MARS 6, CEM, Matthews, NC, USA), using the following digestion program: (1) ramp to reach 120 °C and holding for 10 min; (2) ramp to reach 180 °C and holding for 10 min; and (3) ramp to reach 210 °C and holding for 10 min. Before each digestion, the vessels were decontaminated with 50% HNO_3_ + 20% HF + 20% HCl in the microwave digestion system, rinsed with bi-distillate water, and air-dried. At the end of the procedure, after appropriate dilutions with bi-distilled water, the samples were analysed using Inductively Coupled Plasma Optical Emission Spectroscopy (iCAP 6200 DUO, Thermo Scientific, Waltham, MA, USA), and Ca, Fe, K, Mg, P, Na, Cu, Zn, and Se content was determined. A multi-element certified stock solution (Periodic table mix 3 for ICP; Merck, Darmstadt, Germany) was used to prepare the calibration standards for the measurement using ICP-OES. Spinach leaf standard reference material (NIST-1570A, Sigma Aldrich, St. Louis, MO, USA) was analysed to verify the analytical quality of the data (Table S1).

### 2.5. Thiamine and Riboflavin Analysis

A total of 0.4 g of sample was weighted in 100 mL volumetric flasks, and 2 mL of 0.1 N HCl was added, followed by heating in a water bath at 100 °C for 30 min as in [5]. The extraction was carried out according to Hasselmann et al. [30]. Each sample was analysed in triplicate. Thiamine and riboflavin were identified and quantified by means of known solutions of their corresponding standards. The analytical quality of the data was verified through the analysis of a certified reference vegetable sample (BCR^®^-485, Sigma Aldrich, St. Louis, MO, USA) and a reference wholemeal flour sample (BCR^®^-121, Sigma Aldrich, St. Louis, MO, USA) (Table S1).

### 2.6. Determination of Tocols

Tocols were extracted on 0.1 g of a freeze-dried sample, using the method of alkaline hydrolysis of the food matrix [28], which, from the literature, has been proven as the most effective extraction method of some bioactives [12]. The residues were suspended in a n-hexane:isopropyl alcohol solution (99:1 *v/v*). A HPLC Dionex system (Dionex, Sunnyvale, CA, USA), equipped with an Ultimate 3000 Pump, was used. The chromatographic separation of compounds was performed using a 5 µm Luna column, with a silica stationary phase (250 mm × 4.6 mm i.d.) (Phenomenex, Torrance, CA, USA). A n-hexane:ethyl acetate:acetic acid solution (97.3:1.8:0.9 *v/v/v*) was used as the mobile phase, at a flow rate of 1.6 mL/min. Tocols were detected using a RF 2000 spectrofluorimeter (Dionex, Sunnyvale, CA, USA), set at an excitation and emission wavelength of 290 nm and 330 nm, respectively, and identified and quantified through known standard solutions. Data were processed using a Dionex Chromeleon Version 6.6 chromatography system (Dionex, Sunnyvale, CA, USA). Vitamin E activity was expressed as tocopherol equivalent (TE), calculated as in Sheppard et al. [31]. The performances of the analytical method are reported in [28]. The analysis of α-tocopherol from a certified material (fortified breakfast cereal, NIST-SRM 3233, Sigma Aldrich, St. Louis, MO, USA) is shown in Table S1.

### 2.7. Determination of Carotenoids

Carotenoids were extracted by using the saponification method of Fratianni et al. [23], under the conditions already reported for tocols. Compounds were analysed through a normal (for xanthophylls) and a reverse phase HPLC (for carotenes). A HPLC Dionex (Sunnyvale, CA, USA) analytical system, consisting of a 50 µL injector loop (Rheodyne, Idex Health & Science, Northbrook, IL, USA) and an Ultimate 3000 pump system, was used. For the reverse phase, the mobile phase was methanol:methylterbutylether:water, at a flow rate of 1 mL/min., under a gradient profile as in Mouly et al. [32], by using a 5 µm C30 YMC (Hampsted, NC, USA) stainless steel column (250 × 4.6 mm i.d.). Samples were suspended in methanol/methylterbutylether 50:50 (*v/v*). Under normal phase conditions, the mobile phase was n-hexane:isopropyl alcohol, in a multilinear gradient elution from 10% (A) to 20% (B) of isopropyl alcohol in n-hexane, as reported elsewhere [23]. A 5 µm Luna column, with a silica stationary phase (250 mm × 4.6 mm i.d.), was used (Phenomenex, Torrance, CA, USA). Data were processed using a Dionex Chromeleon Version 6.6 chromatography system (Sunnyvale, CA, USA). Carotenoids were spectrophotometrically detected at 450 nm. Spectral characteristics and the comparison of retention times with those of available standards were used to identify carotenoids. Known standard solutions were used for carotenoid quantification. Vitamin A activity was expressed as retinol equivalent (RE), calculated as in EFSA [33], considering carotene amounts. The performances of the analytical method, together with recovery tests and the analysis of a reference certified vegetable sample (BCR^®^-485), are reported in [10,34].

### 2.8. Statistical Analysis

For raw leaves and for each cooking treatment, three samples were investigated (biological replicates). Each sample was analysed in triplicate (technical replicates). Results are reported as the average of three determinations on three samples. Statistical data are expressed as mean ± standard deviation. An ANOVA test was applied to the data, using a Statistical Software Package (IBM SPSS Statistic 26 for Windows) (SPSS Inc., Chicago, IL, USA). Statistical significance was attributed to *p* values < 0.05 and was obtained by using a least significant difference (LSD) test.

## 3. Results and Discussion

### 3.1. Levels of Minerals

All vegetables were a good source of nutritional minerals (Table 1). Amounts of mineral constituents in vegetable leaves could be variable, depending on the species, environmental conditions, location, and parameters of the applied thermal processing [3,18,29]. Despite the variability, potassium was considerably higher than sodium, as is common in plant foods. The highest levels of K were found in *Sonchus* and spinach leaves (on average, 460 mg/100 g), so 100 g of these vegetables were able to cover 15% of the RDA, and could be declared as a source of this element [35]. The investigated *Sonchus* plants and spinach were a good source of Na (on average, about 130 mg/100 g), while poor levels were found for chicory (1 mg/100 g). With the exception of the lower values of *S. oleraceus*, the mean Ca content was about 70 mg/100 g. P (about 30–70 mg/100 g) and Mg contents (ranges 15–65 mg/100 g) were all in quite good amounts. The levels of trace elements did not exceed 5 mg/100 g, but it is worth noting that 100 g of all plants were able to fulfil 100% of the RDA for Se [35]. The average values were in the range of those found in the literature, even if with slight differences [1,3,4,29]. In particular, in *Sonchus oleraceus,* Na ranged from 44 to 270 mg/100 g, K from 320 to 790 mg/100 g, and Ca from 130 to 230 mg/100 g. For trace elements, Fe ranged from 0.6 to 1.2 mg/100 g and Zn from 0.5 to 0.9 mg/100 g [3]. Similar values were highlighted for *Sonchus asper* for Na (85–211 mg/100 g), K (440–630 mg/100 g), Ca (80–175 mg/100 g), Fe (2–4 mg/100 g), and Zn (0.8–1 mg/100 g) [1]. In the analysed chicory sample, lower Na levels were found compared to the published data (20–270 mg/100 g), while the contents of K and Ca fell within the lower limit of the reported ranges (50–4000 mg/100 g and 45–2000 mg/100 g, respectively). Similar amounts of Fe (0–2.0 mg/100 g) and Zn (0.1–0.5 mg/100 g) were found [1,3,4,29]. Comparing the analysed *Beta vulgaris* sample to a similar genus, such as *Beta maritima* L., the latter showed higher levels of Na and K (45–288 mg/100 g and 597–2356 mg/100 g, respectively) and lower Ca contents (19–25 mg/100 g). Fe and Zn were in the reported ranges (1.4–4.0 mg/100 g and 0.6–1.3 mg/100 g, respectively) [4]. Finally, for spinach, the values from the literature were similar to the investigated ones, about 95, 500, and 65 mg/100 g for Na, K, and Ca, respectively, 1.0 mg/100 for Fe, and 0.3 mg/100 g for Zn [29].

Boiling affected mineral contents (20–60% decreases), with the highest extents for K and Mg (40–60%). Ca losses ranged from 15% in chicory to 50% in *S. asper*; those of P, Fe, Zn, and Se ranged from 30 to 40%. The overall results did not change if expressed on dry weight. In the literature, a different behaviour has been observed for minerals and trace elements during thermal treatments, due to the different forms in which they are present in the plant tissues [29,36]. Notwithstanding losses as to raw samples, 15% of the RDA for Se and K was still covered, respectively, by 100 g and 150 g of the vegetables. The decrease in nutrients during boiling is caused by leaching or diffusion [18]. With the exception of K in spinach and chicory (about 40% losses), steaming did not significantly influence the mineral content (*p* < 0.05).

### 3.2. Content of Carotenoids

The main identified carotenoids were violaxanthin, neoxanthin, lutein, zeaxanthin, β-cryptoxanthin, α-carotene, 13-*cis*-β-carotene, β-carotene, and 9-*cis*-β-carotene. Table 2 reports the amounts of the individual and total carotenoids (TC), expressed as mg/100 g of fresh weight (f.w.), in fresh, boiled, and steamed samples. The amounts of carotenoids found refer to total compounds (free, esterified, and linked to the food matrix), due to the method of alkaline hydrolysis of the food matrix and solvent extraction used.

In all analysed plants, both fresh and cooked ones, the two carotenoids at the highest amounts were lutein and β-carotene. In particular, in raw samples, lutein ranged from about 6 to 9 mg/100 g f.w (on average, 50% of TC), and β-carotene from about 2 to 5 mg/100 g f.w. (on average, 18% of TC in all samples, with the exception of chicory where it was about 33% of TC). There is no defined dietary reference intake for lutein; however, a consumption of 6 to 14 mg of lutein per day has been proposed to lower the risk of macular degeneration and cataract [37]. Therefore, a daily consumption of two portions (160 g) [38] of the investigated green vegetables could fulfil an intake ≥ 10 mg/day. Quite high amounts of violaxanthin and neoxanthin were also found, ranging from 0.7 mg/100 g in *S. oleraceus* to 1.6 mg/100 g in *B. vulgaris*, with a combined average contribution of 16% of TC. The overall data are of the same order of magnitude as those found in different green leafy vegetables, even though they were obtained without the use of the alkaline hydrolysis method. Such data are reported for spinach, regarding epoxycarotenoids [39], lutein (from 0.2 to 8.7 mg/100 g), and β-carotene (from 0.02 to 4.6 mg/100 g) [9,40,41], chard (about 2 mg/100 g of lutein and β-carotene) [40], and chicory, for lutein, β-carotene (in the ranges of 1.8–8.4 mg/100 g and 1.0–4.9 mg/100 g, respectively), and expoxycarotenoids [40,42]. Similar results are also reported in other studies, where the total compound content was analysed in *Sonchus* [5], spinach, chard [10], and chicory [43].

The epoxycarotenoids violaxanthin and neoxanthin were the main molecules that, after domestic treatment, underwent the highest significant reduction. In particular, after boiling, violaxanthin losses significantly ranged from about 20% (*S. asper*) to 90% (*B. vulgaris*), while significant decreases in neoxanthin were observed only in *B. vulgaris* (60%). Greater losses were observed after steaming: in particular, violaxanthin was the most susceptible carotenoid, with decreases ranging from about 40% (*Sp. oleracea*) to 100% (*S. asper* and *C. intybus*). Losses of neoxanthin were observed to a lesser extent (up to 60% in *B. vulgaris*). Similar results were obtained when data were expressed on dry weight, considering solid losses, as in Fratianni et al. [23]. The high susceptibility of epoxycarotenoids to thermal treatment has been proven in vegetable foods [44]. 

Considering the other investigated carotenoids, boiling did not cause significant decreases (*p* < 0.05). On the contrary, a small increase was observed, in some plants, for zeaxanthin and α-carotene. In our case, the complete extraction on the investigated compounds due to the alkaline hydrolysis of the food matrix excluded the increment of their extractability due to heat treatment. As already demonstrated by the authors in a recent paper [23], the leaching of soluble solids after domestic cooking could be responsible for their apparent gain in cooked vegetables. Therefore, considering solid losses, compounds were not statistically affected by boiling, if data were expressed on dry weight [23]. Steaming caused a slight significant decrease in β-carotene, in almost all vegetables (on average, about 20%), and lutein, only in *S. asper* (about 20%) and *Sp. oleracea* (about 10%). Data are in accordance with some studies in which losses were lower during boiling than under other domestic treatments, like steaming [14,23,26]. This behaviour could be ascribed to the activities of some oxidative enzymes that may affect compounds early in the process, where thermal conditions were not sufficient for their inactivation [26,45]. No increases in *cis* isomers were found under any of the applied domestic treatments [22,41]. The loss of epoxycarotenoids caused a corresponding slight significant decrease in total carotenoids, after steaming, in all vegetables (Table 2). From the literature, the effect of different food treatments on carotenoids is difficult to evaluate, due to the various process parameters that could affect their levels, such as temperature, heating time, oxygen, light, pH, and the type of food matrix [14,15,16,17,19], but, also, due to the different analytical procedures carried out for their determination [23]. 

**Figure 1:**
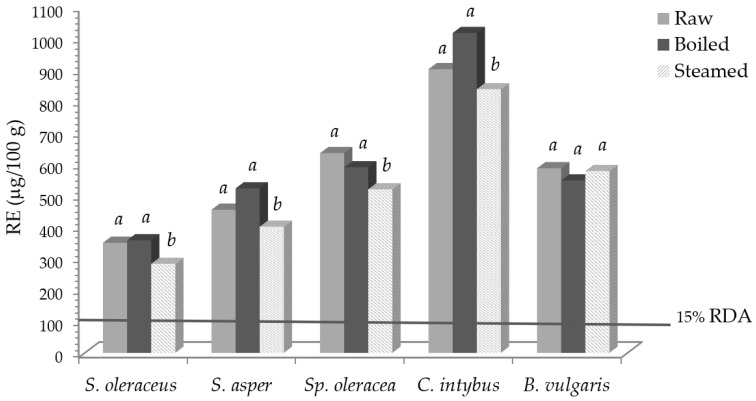
Vitamin A activity (RE) in leafy vegetables before and after domestic cooking and the expressed percentage of the RDA. Different letters in the same vegetable indicate a statistically significant difference among treatments at *p* < 0.05.

Vitamin A activity (RE) and the required percentage of the recommended daily allowance (RDA), before and after domestic cooking, are reported in Figure 1.

Taking into account values for vitamin A, expressed as retinol equivalent (RE) [33], and its recommended daily allowance (RDA) of 800 μg/day of RE [35], 100 g of fresh *S. oleraceus*, *S. asper*, *Sp. Oleracea*, *C. intybus*, and *B. vulgaris* contributed to 44, 57, 79, 122, and 73% of the RDA, respectively. Notwithstanding the reported slight carotenoid losses, an intake of 100 g of some fresh, boiled, and steamed vegetables was able to cover 15% of the RDA, so, according to [35], they can be declared as “sources of vitamin A”. 

### 3.3. Contents of Tocols and B-Group Vitamins

The contents of single and total tocols, thiamine, and riboflavin, expressed as mg/100 g f.w, are given in Table 3. 

Riboflavin was found at low amounts in all plants. *S. oleraceus*, *S. asper*, spinach, and chicory had good levels of thiamine, while chard had the highest amounts, able to cover 100% of the RDA [35]. Few data are reported on B-complex vitamins in WEPs; however, what was found is quite in accordance with the published literature on some green leafy vegetables [1,5]. Almost a total reduction was observed for both vitamins after boiling, with the exception of chard (40% for B1 and 75% for B2). The main reasons for the reduction in thiamine and riboflavin are ascribed to their water solubility and degradation during thermal processing [18]. 

In all investigated samples only tocopherols were found, as also reported, for similar vegetables, in different papers [1,5,23,43,46]. In particular, α-tocopherol was the main detected compound (on average, 2.2 mg/100 g), representing 79% of total tocopherols in *C. intybus*, 87% in *S. oleraceus* and *S. asper*, and 90% *in Sp. oleracea* and *B. vulgaris*. Beta-tocopherol (β-T) was detected at low amounts, exclusively in *B. vulgaris*, while γ-tocopherol (γ-T) was found in all vegetables, with contents, in raw samples, ranging from 0.2 mg/100 g (*B. vulgaris*) to 0.9 mg/100 g (*C. intybus*). Total tocol content was similar in all investigated plants, with an average of 2.5 mg/100 g. Boiling and steaming did not significantly affect single and total tocol amounts. These findings did not change if data were expressed on dry weight, considering weight losses, as reported in Fratianni et al. [23]. A different behaviour has been observed in the literature for tocols, after thermal treatments, depending on the same factors already cited for carotenoids [12,14,23,46]. 

**Figure 2:**
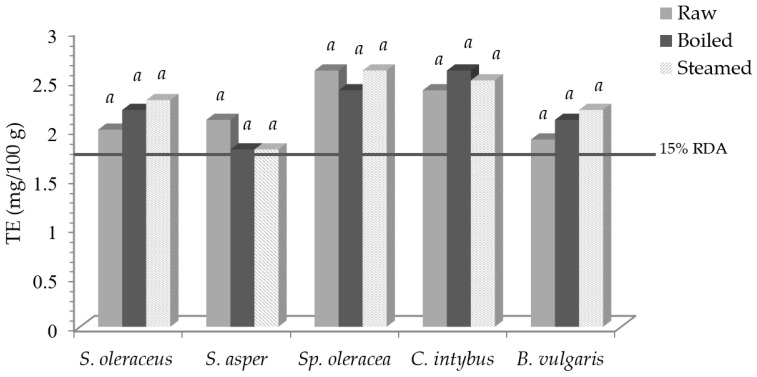
Vitamin E activity (TE) in leafy vegetables before and after domestic cooking and the expected percentage of the RDA. Same letters in the same vegetable indicate a not statistically significant difference among treatments at *p* ≥ 0.05.

Figure 2 reports vitamin E activity, expressed as tocopherol equivalent (TE), and the expected percentage of the RDA.

Taking into account the RDA for vitamin E, which is 12 mg/day of TE [35], 100 g of fresh *S. oleraceus*, *S. asper*, *Sp. oleracea*, *C. intybus*, and *B. vulgaris* contributed to 17, 18, 22, 26, and 16% of the RDA, respectively, with not significant variations as to the cooked products. Therefore, an intake of 100 g of fresh, boiled, and steamed samples was able to cover 15% of the RDA, so, according to [35], all vegetables can be claimed to be “sources of vitamin E”.

## 4. Conclusions

The results from this work confirm that the investigated wild vegetables represent a good source of minerals and tocols, mostly in the form of α-tocopherol, and a relevant source of carotenoids, being important contributors of lutein and β-carotene. The amounts of the less studied carotene isomers (α-carotene, 9-*cis*, and 13-*cis*-β-carotene) and of the epoxycarotenoids violaxanthin and neoxanthin are also given. Since some of the investigated WEPs can be declared as a source of vitamins and minerals, they can contribute to the realization of different functional foods. Boiling affects the content of vitamin B1 and B2 and the amount of minerals. Further research should be carried out to find the proper conditions, such as the vegetable/water ratio, to reduce their leaching. The contents of tocols and carotenoids are not significantly affected by boiling, while a slight carotenoid decrease is observed after steaming. Even giving less contribution to total carotenoids, the epoxycarotenoids show a major susceptibility to thermal treatments. This evidence could suggest their use as process and product indicators, to ascertain the impacts of thermal treatments on food and find the proper process parameters to minimize the extent of losses. Considering the overall findings, they could help to establish appropriate additional databases of bioactive compound content in wild plants, either raw or cooked, mainly where the existing knowledge is absent or weak. This can be the case of most published data on liposoluble compounds, like tocols and carotenoids, that could be intimately associated with the vegetable matrix. Without using an extraction procedure able to recover them from the food matrix, data could not refer to their total amounts, giving a wrong estimation of their real content.

## Figures and Tables

**Table 1:** Levels of minerals in raw, boiled, and steamed green leafy vegetables (mg/100 g f.w.).

	Treatment	*S. oleraceus*	*S. asper*	*Sp. oleracea*	*C. intybus*	*B. vulgaris*
Na	Raw	130.9 (5.5) ^a^	159.7 (5.0) ^a^	106.2 (8.4) ^a^	1.1 (0.1) ^a^	14.0 (0.3) ^a^
	Boiled	66.4 (2.7) ^b^	87.2 (2.1) ^b^	60.1 (5.6) ^b^	0.9 (0.1) ^b^	8.4 (0.1) ^b^
	Steamed	132.1 (4.7) ^a^	157.8 (5.2) ^a^	95.0 (6.8) ^a^	1.2 (0.2) ^a^	13.4 (0.3) ^a^
K	Raw	461.3 (11.1) ^a^	393.3 (31.4) ^a^	545.2 (13.8) ^a^	159.4 (11.7) ^a^	242.4 (13.7) ^a^
	Boiled	245.0 (4.8) ^b^	224.2 (3.3) ^b^	197.1 (6.4) ^c^	68.0 (8.5) ^c^	126.9 (4.7) ^b^
	Steamed	449.2 (7.2) ^a^	378.7 (4.4) ^a^	353.5 (11.8) ^b^	98.5 (14.1) ^b^	222.7 (11.3) ^a^
Ca	Raw	24.7 (1.7) ^a^	66.3 (2.9) ^a^	75.1 (7.4) ^a^	69.4 (6.8) ^a^	78.5 (3.2) ^a^
	Boiled	14.7 (0.2) ^b^	31.5 (0.7) ^b^	53.8 (5.2) ^b^	58.3 (5.1) ^b^	42.9 (3.5) ^b^
	Steamed	26.2 (2.3) ^a^	64.2 (1.4) ^a^	77.9 (3.0) ^a^	73.2 (7.0) ^a^	75.9 (3.0) ^a^
Mg	Raw	55.9 (5.5) ^a^	15.5 (1.8) ^a^	66.3 (1.7) ^a^	30.5 (0.3) ^a^	47.8 (0.7) ^a^
	Boiled	23.7 (0.5) ^b^	8.7 (0.2) ^b^	30.0 (1.6) ^b^	18.2 (0.2) ^b^	33.9 (0.3) ^b^
	Steamed	45.4 (0.2) ^a^	15.1 (0.8) ^a^	64.9 (1.9) ^a^	27.3 (0.3) ^a^	46.7 (0.4) ^a^
P	Raw	36.0 (3.5) ^a^	37.7 (2.5) ^a^	69.2 (1.7) ^a^	29.1 (0.1) ^a^	35.3 (0.3) ^a^
	Boiled	22.9 (0.8) ^b^	22.5 (0.7) ^b^	47.0 (1.4) ^b^	21.7 (0.1) ^b^	23.7 (0.2) ^b^
	Steamed	37.1 (0.3) ^a^	34.3 (0.7) ^a^	70.6 (2.5) ^a^	28.2 (0.1) ^a^	35.3 (0.3) ^a^
Fe	Raw	2.5 (0.2) ^a^	3.2 (0.2) ^a^	2.7 (0.2) ^a^	0.6 (0.1) ^a^	1.1 (0.1) ^a^
	Boiled	1.5 (0.0) ^b^	2.1 (0.1) ^b^	1.5 (0.2) ^b^	0.4 (0.0) ^b^	0.6 (0.0) ^b^
	Steamed	2.3 (0.0) ^a^	3.0 (0.0) ^a^	2.6 (0.1) ^a^	0.5 (0.0) ^a^	1.2 (0.1) ^a^
Cu	Raw	0.19 (0.02) ^a^	0.14 (0.01) ^a^	0.17 (0.06) ^a^	0.07 (0.00) ^a^	0.14 (0.01) ^a^
	Boiled	0.12 (0.00) ^b^	0.13 (0.00) ^a^	0.06 (0.00) ^b^	0.06 (0.00) ^a^	0.10 (0.00) ^b^
	Steamed	0.19 (0.01) ^a^	0.13 (0.01) ^a^	0.10 (0.01) ^a^	0.07 (0.00) ^a^	0.14 (0.01) ^a^
Zn	Raw	1.10 (0.21) ^a^	0.93 (0.08) ^a^	1.07 (0.02) ^a^	0.52 (0.07) ^a^	0.98 (0.14) ^a^
	Boiled	0.67 (0.03) ^b^	0.88 (0.03) ^a^	0.62 (0.01) ^b^	0.31 (0.06) ^b^	0.62 (0.05) ^b^
	Steamed	0.93 (0.03) ^a^	0.90 (0.06) ^a^	0.90 (0.02) ^a^	0.49 (0.04) ^a^	0.90 (0.13) ^a^
Se	Raw	0.10 (0.01) ^a^	0.11 (0.01) ^a^	0.83 (0.12) ^a^	0.82 (0.07) ^a^	1.40 (0.13) ^a^
	Boiled	0.04 (0.00) ^b^	0.06 (0.00) ^b^	0.56 (0.05) ^b^	0.63 (0.05) ^b^	0.94 (0.10) ^b^
	Steamed	0.09 (0.00) ^a^	0.12 (0.01) ^a^	0.80 (0.11) ^a^	0.79 (0.07) ^a^	1.32 (0.11) ^a^

For each compound, different letters in the same column indicate a statistically significant difference at *p* < 0.05.

**Table 2:** Carotenoid content in raw, boiled, and steamed green leafy vegetables (mg/100 g f.w.).

Carotenoids	Treatment	*S. oleraceus*	*S. asper*	*Sp. oleracea*	*C. intybus*	*B. vulgaris*
Violaxanthin	Raw	0.7 (0.1) ^a^	1.1 (0.0) ^a^	1.5 (0.1) ^a^	0.8 (0.1) ^a^	1.6 (0.1) ^a^
	Boiled	0.5 (0.0) ^b^	0.6 (0.0) ^b^	1.2 (0.1) ^b^	0.5 (0.1) ^b^	0.1 (0.0) ^b^
	Steamed	0.1 (0.0) ^c^	---	0.9 (0.1) ^c^	---	0.1 (0.0) ^b^
Neoxanthin	Raw	0.8 (0.0) ^a^	0.9 (0.0) ^a^	1.6 (0.1) ^a^	1.1 (0.0) ^a^	1.1 (0.1) ^a^
	Boiled	0.8 (0.0) ^a^	0.8 (0.1) ^a^	1.5 (0.0) ^a^	1.1 (0.1) ^a^	0.4 (0.0) ^b^
	Steamed	0.6 (0.0) ^b^	0.7 (0.0) ^b^	1.4 (0.0) ^b^	0.5 (0.0) ^b^	0.4 (0.0) ^b^
Lutein	Raw	5.7 (0.6) ^a^	6.1 (0.1) ^a^	8.5 (0.4) ^a^	6.1 (0.3) ^b^	8.8 (0.4) ^b^
	Boiled	6.0 (0.0) ^a^	5.5 (0.2) ^a^	7.2 (0.3) ^b^	6.7 (0.2) ^a^	9.5 (0.1) ^a^
	Steamed	5.6 (0.3) ^a^	4.8 (0.2) ^b^	7.7 (0.5) ^b^	6.2 (0.3) ^b^	9.2 (0.1) ^b^
Zeaxanthin	Raw	0.5 (0.0) ^b^	0.6 (0.0) ^b^	0.7 (0.0) ^b^	0.5 (0.0) ^a^	1.1 (0.1) ^a^
	Boiled	0.9 (0.1) ^a^	0.8 (0.0) ^a^	0.8 (0.0) ^a^	0.5 (0.0) ^a^	1.1 (0.2) ^a^
	Steamed	0.7 (0.1) ^a^	0.6 (0.1) ^b^	0.9 (0.1) ^a^	0.5 (0.0) ^a^	1.1 (0.1) ^a^
β-Cryptoxanthin	Raw	nd	nd	nd	0.8 (0.1) ^a^	nd
	Boiled	nd	nd	nd	1.2 (0.2) ^a^	nd
	Steamed	nd	nd	nd	0.9 (0.1) ^a^	nd
α-Carotene	Raw	0.2 (0.0) ^a^	0.3 (0.0) ^b^	0.6 (0.1) ^a^	0.6 (0.1) ^c^	0.4 (0.0) ^a^
	Boiled	0.3 (0.0) ^a^	0.5 (0.0) ^a^	0.7 (0.0) ^a^	1.0 (0.1) ^a^	0.5 (0.1) ^a^
	Steamed	0.3 (0.0) ^a^	0.3 (0.0) ^b^	0.6 (0.1) ^a^	0.8 (0.0) ^b^	0.6 (0.1) ^a^
13-*Cis*-β-carotene	Raw	0.2 (0.0) ^a^	0.1 (0.0) ^a^	0.1 (0.0) ^a^	0.1 (0.0) ^a^	0.5 (0.0) ^a^
	Boiled	0.3 (0.0) ^a^	0.1 (0.1) ^a^	0.1 (0.0) ^a^	0.2 (0.1) ^a^	0.6 (0.1) ^a^
	Steamed	0.2 (0.0) ^a^	0.1 (0.1) ^a^	0.1 (0.0) ^a^	0.1 (0.0) ^a^	0.5 (0.1) ^a^
β-Carotene	Raw	1.8 (0.1) ^a^	2.2 (0.1) ^a^	3.1 (0.6) ^a^	4.7 (0.4) ^a^	2.9 (0.0) ^a^
	Boiled	1.8 (0.2) ^a^	2.4 (0.1) ^a^	2.6 (0.1) ^a^	4.8 (0.1) ^a^	2.6 (0.4) ^a^
	Steamed	1.3 (0.0) ^b^	1.8 (0.0) ^b^	2.3 (0.1) ^b^	4.0 (0.1) ^b^	2.7 (0.2) ^a^
9-*Cis*-β-carotene	Raw	0.2 (0.0) ^a^	0.7 (0.0) ^a^	1.0 (0.1) ^a^	0.9 (0.0) ^b^	0.5 (0.2) ^a^
	Boiled	0.2 (0.0) ^a^	0.9 (0.1) ^a^	1.1 (0.0) ^a^	1.1 (0.1) ^a^	0.6 (0.3) ^a^
	Steamed	0.1 (0.0) ^b^	0.8 (0.1) ^a^	1.1 (0.0) ^a^	1.0 (0.0) ^a^	0.3 (0.1) ^a^
Totals	Raw	10.1 (0.4) ^a^	12.0 (0.2) ^a^	17.1 (0.3) ^a^	15.6 (0.2) ^b^	16.9 (0.1) ^a^
	Boiled	10.8 (0.7) ^a^	11.6 (0.3) ^a^	15.2 (1.4) ^b^	17.1 (0.4) ^a^	15.4 (0.2) ^b^
	Steamed	8.9 (0.3) ^b^	9.1 (0.2) ^b^	15.0 (1.2) ^b^	14.0 (0.2) ^c^	14.9 (0.4) ^b^

For each compound, different letters in the same column indicate a statistically significant difference at *p* < 0.05; nd: not detectable.

**Table 3:** Tocol and B-group vitamin content in raw, boiled, and steamed green leafy vegetables (mg/100 g f.w.).

Compound	Treatment	*S. oleraceus*	*S. asper*	*Sp. oleracea*	*C. intybus*	*B. vulgaris*
α-Tocopherol	Raw	2.0 (0.1) ^a^	2.0 (0.1) ^a^	2.6 (0.1) ^a^	2.3 (0.1) ^a^	2.0 (0.1) ^a^
	Boiled	2.2 (0.2) ^a^	1.7 (0.2) ^a^	2.4 (0.1) ^a^	2.5 (0.1) ^a^	2.1 (0.0) ^a^
	Steamed	2.2 (0.2) ^a^	1.8 (0.0) ^a^	2.6 (0.2) ^a^	2.5 (0.2) ^a^	2.2 (0.1) ^a^
β-Tocopherol	Raw	nd	nd	nd	nd	0.02 (0.00) ^a^
	Boiled	nd	nd	nd	nd	0.03 (0.00) ^a^
	Steamed	nd	nd	nd	nd	0.02 (0.00) ^a^
γ-Tocopherol	Raw	0.3 (0.0) ^a^	0.3 (0.0) ^a^	0.3 (0.0) ^a^	0.9 (0.1) ^a^	0.2 (0.0) ^a^
	Boiled	0.4 (0.0) ^a^	0.3 (0.0) ^a^	0.4 (0.0) ^a^	0.9 (0.1) ^a^	0.3 (0.0) ^a^
	Steamed	0.4 (0.0) ^a^	0.4 (0.0) ^a^	0.4 (0.0) ^a^	0.8 (0.0) ^a^	0.3 (0.0) ^a^
Total tocols	Raw	2.3 (0.1) ^a^	2.3 (0.1) ^a^	2.9 (0.2) ^a^	3.2 (0.1) ^a^	2.2 (0.1) ^a^
	Boiled	2.6 (0.2) ^a^	2.0 (0.2) ^a^	2.8 (0.1) ^a^	3.4 (0.1) ^a^	2.4 (0.2) ^a^
	Steamed	2.6 (0.3) ^a^	2.2 (0.1) ^a^	3.0 (0.1) ^a^	3.3 (0.5) ^a^	2.5 (0.2) ^a^
Thiamine	Raw	0.10 (0.01) ^a^	0.12 (0.01) ^a^	0.02 (0.00) ^a^	tr	1.58 (0.12) ^a^
	Boiled	tr	tr	tr	nd	0.93 (0.08) ^b^
	Steamed	0.11 (0.01) ^a^	0.10 (0.01) ^a^	0.01 (0.00) ^a^	tr	1.69 (0.15) ^a^
Riboflavin	Raw	0.01 (0.00) ^a^	0.01 (0.00) ^a^	0.01 (0.00) ^a^	0.02 (0.00) ^a^	0.04 (0.00) ^a^
	Boiled	tr	tr	tr	tr	0.01 (0.00) ^b^
	Steamed	0.01 (0.00) ^a^	0.01 (0.00) ^a^	0.01 (0.00) ^a^	0.02 (0.00) ^a^	0.05 (0.01) ^a^

For each compound, different letters in the same column indicate a statistically significant difference at *p* < 0.05; nd: not detectable; tr: traces (values ≤ 0.001 mg/100 g).

## Data Availability

Data is contained within the article.

## Supplementary Materials

The following supporting information can be downloaded at: https://www.mdpi.com/article/10.3390/foods13030472/s1, Table S1: Bias (%) for Ca, P, K, Na, Cu, Zn, α-tocopherol, B1 and B2 vitamins as to values of certified reference materials.
