# Supplementary material for: Evaluation of the Content of Minerals, B-Group Vitamins, Tocols, and Carotenoids in Raw and In-House Cooked Wild Edible Plants
Source: Foods. 2024 Feb 2;13(3):472. doi: 10.3390/foods13030472 (PMC10855799; doi:10.3390/foods13030472)
Supplement: Supplementary file 1 [file foods-13-00472-s001.zip › foods-2821253-supplementary.pdf]

Table S1. Bias (%) for Ca, P, K, Na, Cu, Zn, α-tocopherol, B1 and B2 vitamins as to values of certified reference materials.

Compounds	Matrix reference value	Measured value	Bias (%)
Ca (g/100g) *	1.526 ± 0.066	1.494 ± 0.042	-2.14
P (g/100g)	0.519 ± 0.001	0.533 ± 0.012	2,62
K(g/100g)	2.900 ± 0.026	2.770 ± 0.042	-4.69
Na (g/100g)	1.821 ± 0.023	1.829 ± 0.036	0.44
Cu (mg/Kg)	12.22 ± 0.86	12.34 ± 0.45	0.97
Zn(mg/Kg)	82.30 ± 3.90	81.26 ± 1.27	-1.28
α-tocopherol (mg/kg) **	1350.0 ± 220	1450.3 ± 52.1	6.92
B1 (mg/kg d.m.) °	3.07 ± 0.34	2.92 ± 0.15	-5.13
B1 (mg/kg d.m.) §	4.63 ± 0.39	4.36 ± 0.22	-6.19
B2 (mg/kg d.m.) §	0.90 ± 0.05	0.85 ± 0.06	-5.88

* Reference samples for mineral and trace elements: spinach leaves (SRM NIST-1570A); ** fortified breakfast cereal (SRM NIST-3233); ° mixed vegetables (CRM BCR-485); § wholemeal flour (CRM BCR-121): values are certified for B1 and declared for B2. For carotenoids a mixed vegetables reference sample (CRM BCR-485) was analysed (for details see [10,34]). Bias (%) = (measured value-reference value)/measured value X 100; d.m.: dry matter
